# The Evaluation of Retinal Nerve Fiber Layer Thickness in Patients with Obstructive Sleep Apnea Syndrome

**DOI:** 10.1155/2013/292158

**Published:** 2013-11-27

**Authors:** Mehmet Adam, Mehmet Okka, Şebnem Yosunkaya, Banu Bozkurt, Hürkan Kerimoğlu, Meydan Turan

**Affiliations:** ^1^Department of Ophthalmology, School of Medicine, Bozok University, Yozgat, 66000 Konya, Turkey; ^2^Department of Ophthalmology, Meram School of Medicine, Necmettin Erbakan University, Konya, Turkey; ^3^Department of Pulmonary Medicine, Meram School of Medicine, Necmettin Erbakan University, Konya, Turkey; ^4^Department of Ophthalmology, School of Medicine, Selçuk University, Turkey; ^5^Department of Ophthalmology, Susurluk State Hospital, Balıkesir, Turkey

## Abstract

*Aim*. To evaluate the retinal nerve fiber layer (RNFL) thickness in patients with obstructive sleep apnea syndrome (OSAS) by optical coherence tomography (OCT). *Materials and Method*. We studied 43 new diagnosed OSAS patients and 40 healthy volunteers. Patients underwent an overnight sleep study in an effort to diagnose and determine the severity of OSAS. RNFL analyses were performed using Stratus OCT. The average and the four-quadrant RNFL thickness were evaluated. *Results*. There was no difference between the average and the four-quadrant RNFL thickness in OSAS and control groups. There was no correlation between apnea-hypopnea index and intraocular pressure. Body mass index of patients with moderate and severe OSAS was significantly higher in patients with mild OSAS. *Conclusion*. Mean RNFL thickness did not differ between the healthy and the OSAS subjects, however, the parameters were more variable, with a larger range in OSAS patients compared to controls.

## 1. Introduction

Obstructive sleep apnea syndrome (OSAS) is a syndrome which is characterized by recurrent partial or complete upper airway obstruction during sleep [1]. OSAS is a common sleep disorder that can be seen in both sexes and all races, ages, socioeconomic statuses, and ethnic groups. According to the data reported in the United States, in addition to the symptom of sleepiness, polysomnography (PSG) performed in adult men and women between the ages of 30 and 60 was diagnosed with OSAS rates of 4% and 2%, respectively [2]. In Turkey, a study conducted by Köktürk et al. showed that the prevalence of OSAS in men and women is 1.9% and 0.9%, respectively [3]. The risk factors for OSAS are obesity, male gender, thick neck, upper respiratory tract abnormalities, genetic predisposition, smoking, and alcohol use [4]. Frequently reported symptoms of OSAS are loud snoring, excessive daytime sleepiness, waking up in the morning tired, and morning headache [1]. For patients with loud snoring and excessive daytime sleepiness, polysomnography is recommended. Otherwise it is not recommended to those without these complaints [5].

The combination of OSAS with glaucoma was first drawn to our attention in 1982 by Walsh and Montplaisir's study which reported that 5 members of the same family had OSAS with glaucoma [6]. While there are studies reporting high glaucoma prevalence or decrease in retinal nerve fiber layer (RNFL) thickness in patients with OSAS, there are also studies arguing that OSAS does not have any relationship with glaucoma [7–17].

Optic neuropathy associated with glaucoma is characterized by increased size of optic nerve head cup/disc ratio and thinning of the retinal nerve fiber layer [18]. In a study assessing the progress of patients with glaucoma and suspicion of glaucoma, thinning RNFL thickness has been observed in 22% of the patients with optical coherence tomography (OCT) without any progress in the vision field [19]. The early detection of thinning in RNFL increases the chance of early diagnosis of glaucoma [18].

In this study, we evaluated RNFL thickness in new diagnosed OSAS patients with corrected intraocular pressure (IOP) below 21 mmHg.

## 2. Materials and Methods

The current study was carried out in Necmettin Erbakan University, Meram Medical Faculty, Departments of Ophthalmology and Chest Disease, between June 2008 and December 2009. This study included 43 patients who were newly diagnosed as OSAS by polysomnography and 40 healthy control subjects. 

Overnight prospective polysomnography was undergone with a VIASYS Sleep Screen System (VIASYS Healthcare GmbH, Hoechberg, Germany). For each patient, we obtained an electroencephalogram (3 channels: C3A2, C4A1, and O2A1), left and right electrooculograms, and a chin electromyogram from surface leads (attached to collect data on sleep state). We used a nasal cannula to measure air flow, thoracic and abdominal strain gauges to quantify respiratory effort, a tracheal microphone to record snoring and a pulse oximeter to measure oxyhemoglobin level, and a sensor to collect information on body position during sleep. The control group was composed of volunteers who had no snoring, witnessed apneas, gasping/choking episodes, excessive sleepiness not explained by other factors, and OSAS according to examination conducted by the pulmonologist.

Apnea-hypopnea index (AHI) is calculated by dividing the number of apneas and hypopneas by sleep time. Patients were classified according to AHI. Those with AHI of 6–15, 16–30, and 31 or above were considered as mild, moderate, and severe OSAS patients, respectively. All patients underwent chest disease and ocular examination. The best corrected visual acuity of all subjects was recorded according to Snellen chart. The central corneal thickness of subjects was measured by the Pentacam Scheimpflug (Oculus, Inc., Wetzlar, Germany) before dilation of pupil. Ehlers formula was used for corrected IOP [20]. After measuring IOP with Goldmann applanation tonometry, gonioscopy examinations were performed by Goldmann three-mirror lens. Visual field examination was performed by Humphrey Field Analyzer 750i (Humphrey Systems, Inc., Dublin, CA, USA). After pupil dilation, fundus examination was performed.

Subjects who had intraocular surgery, corrected IOP higher than 21 mmHg, diabetic retinopathy, and refractive error on 2D and patients with glaucoma history, ocular trauma, and uveitis history were excluded. Moreover, patients who had signs of peripapillary choroidal atrophy and abnormal ophthalmoscopic examination of the optic nerve head, macula, and retinal vasculature were also excluded. 

The RNFL analyses were performed using Stratus OCT-3 (Carl Zeiss Meditec, Inc., CA, USA). Measurement was performed according to the “Fast RNFL protocol” between the hours of 8:30 and 9:30 a.m. by the same physician (M. Adam) Measurement was taken in triplicate for each eye. The best provided signal strength higher than 7 was used for analysis. The average RNFL thickness and the four-quadrant (superior, nasal, inferior, and temporal) RNFL thickness were determined as micron (*μ*). 

For statistical analysis, Statistical Package for Social Science (SPSS) program (Worldwide Headquarters SPSS, Inc. 15.0 Windows package program) was used. Descriptive findings are displayed as mean ± standard deviation. Normal distribution of the data was assessed according to Kolmogorov-Smirnov test in both groups. Comparisons between groups were analyzed via Student's *t*-test; one-way ANOVA was used for multiple comparisons between groups. Chi-square test was used to compare categorical variables. Pearson's correlation test was used for correlation analysis. *P* values < 0.05 were considered statistically significant.

## 3. Results

Forty-three patients with OSAS (4 mild, 23 moderate, and 16 severe) and forty healthy volunteers were studied. There was no difference between OSAS and control groups with respect to age, gender, visual acuity, IOP, central corneal thickness, and refractive values (Table 1).

**Figure 1:**
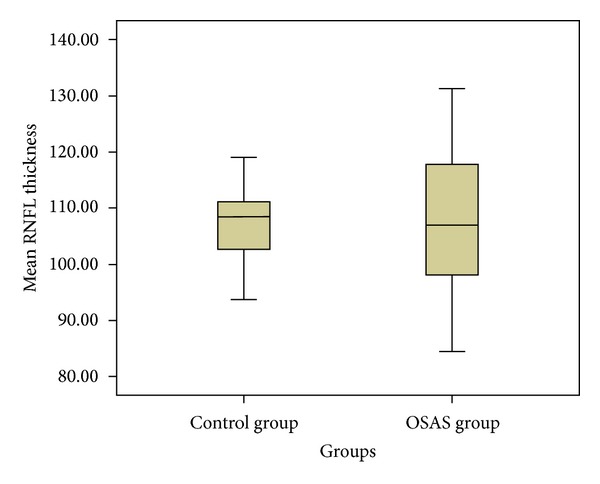
The distribution of average retinal nerve fiber layer thickness (RNFL) thickness in Obstructive sleep apnea syndrome (OSAS) and control groups.

The mean RNFL thickness ranged between 84.45 and 131.25 *μ* in patients with OSAS and 93.70 between 119 *μ* in controls, respectively. For RNFL thickness of quadrants in patients and controls, the following were found: superior: 52–165 *μ*, 97–162 *μ*; inferior: 86–200 *μ*, 115–164 *μ*; nasal 56–127*μ*, 54–115 *μ*, and temporal: 53–123 *μ*, 53–113 *μ*, respectively.  Figure 1 shows the distribution of average RNFL thickness in patients and control group. The mean RNFL thicknesses in the groups were summarized in Table 2.

When compared according to OSAS stages and average saturation level of patients during sleep, no difference was found between the patients and the control group (Tables 3 and 4).

When central visual field analyses (30-2) of OSAS and control groups were compared, the mean deviation values were found between −1.21 ± 1.61 and −0.90 ± 1.04, and pattern standard deviation values were found between 2.24 ± 0.64 and 2.31 ± 0.86, respectively. However, there was no significant difference between the groups (resp., *P* = 0.10, 0.13). Body mass index (BMI) was found to be 27.98 ± 1.35 in patients with mild OSAS 31.41 ± 4.53 in patients with moderate OSAS and 32.93 ± 4.41 in patients with severe OSAS. There was a significant difference regarding BMI between the mild OSAS moderate and severe OSAS patients (*P* = 0.045, 0.001, resp.) but no correlation was found between AHI and corrected IOP (*P* = 0.31,  *r* = 0.17).

## 4. Discussion

Due to recurrent airway obstruction in OSAS, the blood oxygen saturation decreases and hypercapnia occurs. Two possible mechanisms have been speculated for thinning of the RNFL in patients with OSAS. 

Deterioration of autoregulation in blood flow to the optic nerve was due to recurrent apneas, and optic nerve blood flow dysregulation was due to OSAS [21]. In this mechanism, mediators that cause dilatation or contraction of smooth muscle are secreted from normal endothelium tissue. It has been suggested that the balance of endothelial and nitric oxide is disturbed in patients with OSAS. Kato et al. have shown that, in the patients with OSAS, infusion of acetylcholine reduced vascular dilatation due to endothelium-derived nitric oxide and therefore blood flow, compared with control group. However, there was no difference between the groups in endothelium-independent vasodilatation [22]. 

Another speculated mechanism for thinning of the RNFL in patients with OSAS is that vasodilation caused by hypoxia and hypercapnia increases intracranial pressure and, therefore, indirectly disturbs cerebral perfusion and the blood flow to the optic nerve. It has been shown that, in patients with OSAS, intracranial pressure was increased by apnea episodes occurring during sleep; however, it was found to be normal in those patients during the day [23]. In addition, in a study where ophthalmic artery resistance and central artery resistance were examined in patients with OSAS by Doppler ultrasonography, it was found that there is no significant difference in vascular resistance and intraocular pressure however, there was a positive correlation between AHI and IOP; nonetheless, no difference was between IOP of the two groups [24]. Erdem et al. reported that they measured postsystolic and enddiastolic volumes by Doppler ultrasonography in patients with severe OSAS; the blood flow was significantly increased in ophthalmic artery, in central retinal artery, and in posterior ciliary artery, whereas in patients of mild OSAS the blood flow increased only in posterior ciliary artery [25]. This increase may be a compensatory response occurring against chronic hypoxia.

Kergoat et al. reported that the retinal ganglion cells are particularly sensitive to normal perfusion and a decrease in oxygen saturation [26]. In the same study, despite the continuation of systemic hypoxia, electrophysiological parameters showed recovery in later stages of the test.

While there are studies that relate OSAS with glaucoma [7–13] there are also other studies that disagree with this relationship [14–17]. Sergi et al. found that the prevalence of normotensive glaucoma is 5.9% in 51 patients with OSAS [7]. In our study, we did not observe normotensive glaucoma in patients with OSAS. This difference could be because of difference in the mean age of patients included in the studies. The mean age of OSAS patients was 48 ± 7.97, 64 ± 10 in our study, while it was 64 ± 10 in the study by Sergi et al. Moreover, they detected IOP to be significantly higher, albeit within normal limits, for the OSAS patients in comparison to the control group.

In a study conducted by Marcus et al., 23 patients with normotensive glaucoma and 14 patients, with suspected normotensive glaucoma were examined [8]. In this study, OSAS was detected in five patients and hypopnea syndrome was detected in two patients with normotensive glaucoma. However, in Marcus et al.'s study, five of the seven patients diagnosed with normotensive glaucoma and sleep disturbances had diabetes mellitus. Diabetes could be a factor for increasing hypoxia. Gönül et al. found that patients with diabetic retinopathy had significantly lower RNFL values than the control group [27]. In our study, there was only one patient who had diabetes, but not retinopathy. In addition, Marcus et al. took 24 mmHg as threshold for normotensive glaucoma, and they did not assess central corneal thickness. In our study, OSAS patients whose IOP was over 21 mmHg were not included in the study, and we considered the corrected IOP of the patients.

Kargi et al. found that the RNFL thickness decreased in correlation with the severity of OSAS in a study on OSAS patient with NFA GDx device [9]. Lin et al. reported that RNFL thickness of the patients is thinner by using Stratus OCT device and RNFL thinning was correlated with the severity of OSAS [13]. In our study, no difference was found between the OSAS and the control groups in RNFL thickness when compared both in terms of AHI and oxygen saturation. Our study is limited by small sample size of subjects. We could not find a statistically significant difference with a lower number of samples. However, it is questionable whether or not the statistical significance which would be obtained by increasing the sampling size is of clinical importance. Yet one of the results of our study is remarkable. While there was no difference between the OSAS patients and the control group as to their thickness of RNFL, the distribution of the RNFL thickness ranged across a wider band for the OSAS patients (Figure 1). The RNFL thickness ranged between 84.45 and 131.25 *μ* for the OSAS patients; this range was detected as 93.70–119.01 for the control group. The number of cases with RNFL thickness over 120 *μ* is 9 in the OSAS patients, and no case with RNFL thickness over 120 *μ* was detected in the control group. Besides, in the OSAS group, 12 cases had RNFL thickness below 100 *μ*, and the control group had 5 such cases. This finding brings to mind the following questions. Can the reiterating and continuing nighttime hypoxia lead first to edema and therefore high measurement of RNFL thickness and later to damage in the RNFL as a result of chronic hypoxia? It is already known that acute ischemia first leads to edema in the neurons and to degeneration in the postischemic period [28]. Are the measured values higher than the actual figures due to edema? Teramot et al. illustrated that the serum vascular endothelial growth factor (VEGF) level is at maximum after sleep and decreases by oxygen administration for the OSAS patients [29]. It is known that VEGF increases the vascular permeability [30]. May this be the reason why the RNFL thickness values of some cases in the OSAS group were higher than expected? In order to answer these questions, further study is needed for examination of the relationship between serum VEGF levels and the RNFL thickness.

Geyer et al. found that the prevalence of glaucoma is similar in 228 patients with OSAS and normal subjects [14]. Moreover, they could not find any correlation between AHI and glaucoma. However they detected a positive correlation between VKI and AHI. Also, in our study, we could not find any difference between the OSAS group and the control group in terms of their RNFL thickness. We found positive correlation between VKI and AHI. 

Girkin et al. suggested that the sleep apnea is not a risk factor for glaucoma in 667 patients newly diagnosed with glaucoma [15]. Similarly, there are also studies showing the same prevalence of normotensive glaucoma in normal subjects and in patients with OSAS [16, 17].

Roberts et al. have shown that there was no difference between the control group and patients with glaucoma during the night, in terms of oxygen saturation [31]. However, Mojon et al. found that the oxygen desaturation index of 30 patients with primary open-angle glaucoma is higher during sleep than that of the control group. When we compare these two studies, it can be observed that Robert et al. researched on more patients than Mojon et al. (resp., 42 and 30), and the patients in Mojon et al.'s study are older than patients in Robert et al.'s study (76 ± 7.9 and 71 ± 9.0, resp.). These findings bring to mind the question whether the oxygen desaturation occurring during sleep might need a longer time to result in damage in saturation. In a later study, Mojon et al. did not encounter normotensive glaucoma in patients with OSAS and aged less than 45, and they wrote that 63% of the patients over 65 and with normotensive glaucoma were OSAS patients as well [11]. Nevertheless they carried out this research on a limited number of patients and detected only six OSAS cases. In a study carried out on 100 patients with OSAS Bendel et al. found glaucoma frequency higher than expected; 27%. Bendel et al. found glaucoma frequency unrelated to gender, VKI and AHI but related to age [12]. In our study we had only three patients over 60 and the eldest patient was 66 years old. The reason why no difference was detected when RNFL thickness was compared to the control group might result from the fact that the exposition duration to chronic hypoxia during sleep is short. 

## 5. Conclusion 

Although RNFL thickness was not different between the control and OSAS groups, it was more variable in patients compared with controls, possibly because of retinal edema occurring in the early stages of the disease. Duration of the OSAS may be more important than its severity. A long-term follow-up study in the same group of OSAS patients can help in providing answers to these questions.

## Figures and Tables

**Table 1:** Demographic characteristics of obstructive sleep apnea syndrome (OSAS) and control groups.

	OSAS group (*n* = 43)	Control group (*n* = 40)	*P*
Age (year)	48.42 ± 7.97	52.90 ± 8.02	0.07
Sex (*n*, %)			
Female	9 (20.9)	13 (32.5)	
Male	34 (80.1)	27 (67.5)	0.16
Visual acuity (Snellen)	0.99 ± 0.10	1.00 ± 0.0	0.19
IOP (mmHg)	13.90 ± 2.99	14.52 ± 2.76	0.11
Refraction (D) (SE)	−0.12 ± 0.87	0.27 ± 1.11	0.82
CCT (*µ*)	551.72 ± 31.70	548.82 ± 25.21	0.65

SE: spheric equivalent, D: diopter, IOP: intraocular pressure, and CCT: central corneal thickness.

**Table 2:** The mean retinal nerve fiber layer (RNFL) thickness (*µ*) values in the groups.

RNFL thickness, *µ*	OSAS (*n* = 43)	Control (*n* = 40)	*P*
Superior	130.23 ± 14.89	131.33 ± 14.24	0.73
Nasal	83.07 ± 16.76	79.83 ± 15.65	0.37
Inferior	138.84 ± 25.95	140.65 ± 11.18	0.68
Temporal	79.19 ± 14.71	78.45 ± 14.25	0.82
Average RNFL	108.05 ± 12.36	107.54 ± 6.07	0.81

**Table 3:** RNFL thickness (*µ*) based on stages of OSAS.

	Superior	Nasal	Inferior	Temporal	Mean
Mild OSAS	132.50 ± 12.9	88.25 ± 8.2	154.50 ± 18.7	84.00 ± 13.3	114.90 ± 13.3
Moderate OSAS	132.50 ± 15.8	79.94 ± 16.5	127.56 ± 28.1	75.56 ± 11.3	104.46 ± 13.2
Severe OSAS	128.26 ± 14.8	84.35 ± 18.1	143.96 ± 21.0	80.87 ± 17.1	109.36 ± 11.3

*P*	0.83	0.58	0.08	0.48	0.19

OSAS: obstructive sleep apnea syndrome.

**Table 4:** According to average oxygen saturation RNFL thickness (*µ*) of patients with OSAS (cut off value 90%).

	Superior	Nasal	Inferior	Temporal	Average
Group 1	126.13 ± 7.93	83.40 ± 16.52	142.86 ± 26.67	79.53 ± 14.27	107.68 ± 12.50
Group 2	132.43 ± 17.3	83.43 ± 17.18	136.68 ± 25.78	79.00 ± 15.19	108.26 ± 12.51

*P*	0.19	0.85	0.46	0.91	0.89

OSAS: obstructive sleep apnea syndrome.

Group 1: average oxygen saturation ≤ 89%, *n* = 15 (34.89%).

Group 2: average oxygen saturation > 90%, *n* = 28 (65.11%).
